# Darker eggs of mosquitoes resist more to dry conditions: Melanin enhances serosal cuticle contribution in egg resistance to desiccation in *Aedes*, *Anopheles* and *Culex* vectors

**DOI:** 10.1371/journal.pntd.0006063

**Published:** 2017-10-30

**Authors:** Luana C. Farnesi, Helena C. M. Vargas, Denise Valle, Gustavo L. Rezende

**Affiliations:** 1 Laboratório de Biologia Molecular de Insetos, Instituto Oswaldo Cruz, Fiocruz, Rio de Janeiro, RJ, Brazil; 2 Laboratório de Química e Função de Proteínas e Peptídeos, Centro de Biociências e Biotecnologia, Universidade Estadual do Norte Fluminense Darcy Ribeiro, Campos dos Goytacazes, RJ, Brazil; 3 Laboratório de Biologia Molecular de Flavivírus, Instituto Oswaldo Cruz, Fiocruz, Rio de Janeiro, RJ, Brazil; 4 Instituto Nacional de Ciência e Tecnologia em Entomologia Molecular, Rio de Janeiro, RJ, Brazil; Universidade Federal do Rio de Janeiro, BRAZIL

## Abstract

Mosquito vectors lay their white eggs in the aquatic milieu. During early embryogenesis water passes freely through the transparent eggshell, which at this moment is composed of exochorion and endochorion. Within two hours the endochorion darkens via melanization but even so eggs shrink and perish if removed from moisture. However, during mid-embryogenesis, cells of the extraembryonic serosa secrete the serosal cuticle, localized right below the endochorion, becoming the third and innermost eggshell layer. Serosal cuticle formation greatly reduces water flow and allows egg survival outside the water. The degree of egg resistance to desiccation (ERD) at late embryogenesis varies among different species: *Aedes aegypti*, *Anopheles aquasalis* and *Culex quinquefasciatus* eggs can survive in a dry environment for ≥ 72, 24 and 5 hours, respectively. In some adult insects, darker-body individuals show greater resistance to desiccation than lighter ones. We asked if egg melanization enhances mosquito serosal cuticle-dependent ERD. Species with higher ERD at late embryogenesis exhibit more melanized eggshells. The melanization-ERD hypothesis was confirmed employing two *Anopheles quadrimaculatus* strains, the wild type and the mutant GORO, with a dark-brown and a golden eggshell, respectively. In all cases, serosal cuticle formation is fundamental for the establishment of an efficient ERD but egg viability outside the water is much higher in mosquitoes with darker eggshells than in those with lighter ones. The finding that pigmentation influences egg water balance is relevant to understand the evolutionary history of insect egg coloration. Since eggshell and adult cuticle pigmentation ensure insect survivorship in some cases, they should be considered regarding species fitness and novel approaches for vector or pest insects control.

## Author summary

Mosquitoes transmit various causative agents of diseases and the blockage of vector life cycle is an effective way to hamper disease transmission. The egg is the least known life stage and understanding it can contribute with novel strategies for mosquito control. Mosquitoes lay eggs in water collections, some of which are temporary. At early embryogenesis eggs are prone to lose water, leading to dehydration and death. During embryogenesis the serosal cuticle is produced, it wraps the embryo and contributes to the egg protection, allowing it to survive outside the water. Curiously, this resistance varies among mosquitoes: *Aedes*, *Anopheles* and *Culex* eggs can survive outside the water for long, intermediate and short periods, respectively. Here, we show that these differences are related to the degree of eggshell melanization (melanin is a dark pigment): darker eggs resists more against water loss. We confirmed that melanin increases survival outside the water employing a mosquito mutant that does not melanize properly. The protection conferred by melanin is dependent on the formation of the serosal cuticle. Our results contribute to the study of the evolution of egg coloration in insects and we identified one of the reasons why *Aedes aegypti* eggs survive for several months outside water.

## Introduction

Mosquitoes of the genera *Aedes*, *Anopheles* and *Culex* transmit pathogens that are the causative agents of diverse diseases such as yellow fever, dengue, chikungunya, Zika and West Nile viruses, malaria and lymphatic filariasis [1–7]. Blocking mosquito life cycle is an effective way to hamper disease transmission [8].

**Fig 1:**
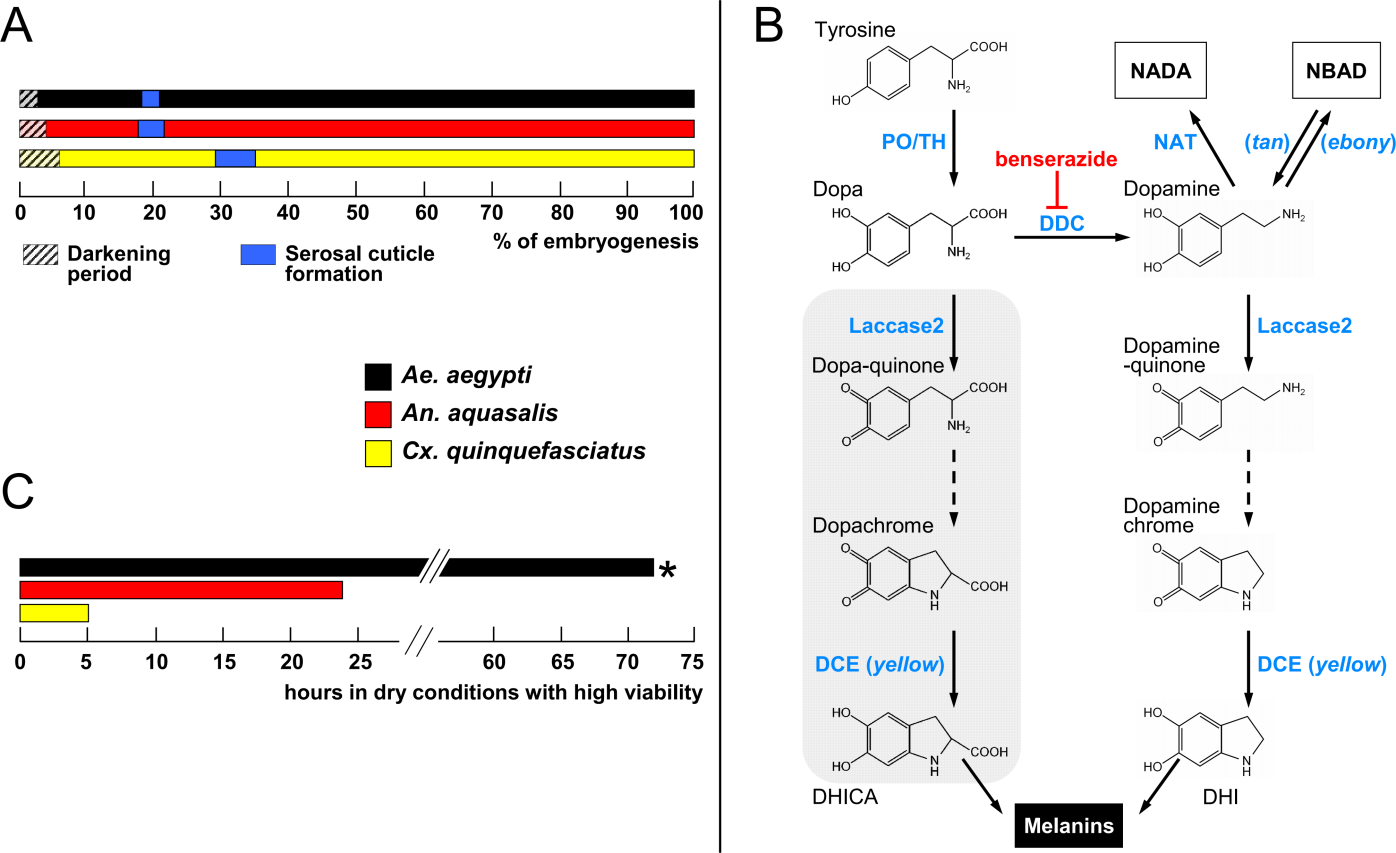
Events related to mosquito embryogenesis. (**A**) Periods of egg darkening and serosal cuticle formation. Data are shown as percentages of the total embryonic development for each species, which is 77.4, 51.3 and 34.2 hours after egg laying for *Ae*. *aegypti*, *An*. *aquasalis* and *Cx*. *quinquefasciatus*, respectively. (**B**) Melanization pathway. Chromes are formed non-enzymatically (dashed arrows). DHICA: 5,6-dihydroxyindole-2-carboxylic acid, DHI: 5,6-dihydroxyindole. NADA (N-acetyldopamine) and NBAD (N-β-alanyldopamine) are also substrates for Laccase 2, originating quinones that participate in the sclerotization pathway. Grey background: Dopa contribution for melanin formation is minor since it is a poor substrate for Laccase2 (see main text). Enzyme names are shown in blue and *Drosophila melanogaster* mutants, in italic. PO: phenoloxidase, TH: tyrosine hydroxylase, DCE: dopachrome conversion enzyme, DDC: dopa decarboxylase, NAT: N-acetyltransferase, *tan*: N-β-alanyldopamine hydrolase, *ebony*: N-β-alanyldopamine synthase. Red inhibition symbol: the drug benserazide inhibits DDC activity. (**C**) Egg resistance to desiccation at the end of embryogenesis. At 80% of total embryogenesis, eggs were transferred from water to dry conditions (20–55% relative humidity), and their viability monitored at regular intervals. *Ae. *aegypti* eggs are viable outside water for even longer periods, up to several months [1,17,21]. All data in **A** and **C** were recovered from Vargas *et al*. [19], except darkening period obtained from Christophers [1] and Clements [2].

Mosquitoes lay their eggs in water pools, some of which are temporary [2]. Water passes freely through their eggshells during early embryogenesis and drying these water collections leads to egg desiccation, preventing its development. At this stage mosquito eggshell is composed of a brittle exochorion and a smooth transparent endochorion [2,9]. Laid eggs are white and their endochorion darkens less than three hours after being laid [1,2], (Fig 1A) due to the process that produces eumelanin, a brown to black pigment [10]. Throughout this work the eumelanin will be simply named "melanin".

Melanization commences with L-tyrosine hydroxylation driven by phenoloxidase or tyrosine hydroxylase (Fig 1B). The resulting dopa is decarboxylated via dopa decarboxylase (DDC) giving rise to dopamine. Laccase 2 acts upon both dopa or dopamine oxidizing them and forming quinones that are further cyclized non-enzymatically originating dopachrome or dopaminechrome. These two molecules are substrates for dopachrome conversion enzyme (DCE, also known as *yellow* in *Drosophila melanogaster*) originating DHICA (5,6-dihydroxyindole-2-carboxylic acid) and DHI (5,6-dihydroxyindole) that are further employed in the synthesis of the polymeric melanin. Since dopa is an inadequate substrate for Laccase2 its contribution for melanin formation is minor. Dopamine can also be β-alanylated or acetylated, originating NBAD (N-β-alanyldopamine) or NADA (N-acetyldopamine) that are further transformed into quinones that participate in sclerotization (Fig 1B) [11–16].

However, even melanized *Aedes* eggs shrink and die in a few hours if removed from a moisten environment [16,17]. On the other hand, between 17 and 35 percent of embryogenesis (this percentage varies among species), the serosa, an extraembryonic membrane, secretes the serosal cuticle (Fig 1A). The serosal cuticle is an extracellular matrix; located below the endochorion, becomes the third and innermost eggshell layer. Its formation considerably reduces water passage through the eggshell, preventing eggs to shrink due to desiccation and prompting eggs to maintain their viability outside the water [17,18].

Curiously, the level of egg resistance to desiccation (ERD) varies among mosquito species at the end of embryogenesis: while *Aedes aegypti* eggs can survive for at least 72 hours in a dry environment (high ERD), those of *Anopheles aquasalis* and *Culex quinquefasciatus* under the same condition can survive, respectively, for 24 hours (medium ERD) and 5 hours (low ERD) (Fig 1C) [19]. Physical and biochemical features of these eggs were investigated in order to identify traits associated with these differences. Chitin content is directly related to ERD levels while both egg volume increase during embryogenesis and eggshell superficial density are inversely related to it. Moreover, other yet unidentified traits might also be relevant [20].

Although melanization increases the desiccation resistance of adult insects of different orders [22–25] it is currently unknown if the same process occurs in insect eggs. We investigated here if the intensity of eggshell pigmentation is associated with the levels of desiccation resistance in mosquito vector eggs.

## Methods

### Mosquito sources and rearing

Experiments were conducted with *Aedes aegypti* (Linnaeus, 1762), *Anopheles aquasalis* (Curry, 1932) and *Culex quinquefasciatus* (Say, 1823) continuously maintained at the Laboratório de Fisiologia e Controle de Artrópodes Vetores (LAFICAVE), Instituto Oswaldo Cruz, Rio de Janeiro, RJ, Brazil. The strains ORLANDO and GORO of *Anopheles quadrimaculatus* (Say, 1824) were reared between March and August 2013 at the Florida Medical Entomology Laboratory (FMEL), Florida University, Vero Beach, FL, USA. Both *An*. *quadrimaculatus* strains, ORLANDO (MRA-139) (https://www.beiresources.org/Catalog/BEIVectors/MRA-139.aspx - acessed 15 February 2016) and GORO (MRA-891) (https://www.beiresources.org/Catalog/BEIVectors/MRA-891.aspx - accessed 15 February 2017) were obtained through the Malaria Research and Reference Reagent Resource Center (MR4) (Manassas, VA, USA), as part of the Biodefense and Emerging Infections Research Resources Repository (BEI Resources), NIAID, NIH and were deposited by MQ Benedict. The *An*. *quadrimaculatus* ORLANDO strain is mentioned in this work as "WT" (i.e. wild type). The *An*. *quadrimaculatus* GORO strain contains two independent EMS-induced mutations, both on the X chromosome, and was generated by crossing the GOCUT strain (MRA-123, containing the *golden cuticle* phenotype) and the ROSEYE strain (MRA-122, containing the *rose eye* color phenotype); hence the name GORO: GOlden cuticle + ROse eyes. GORO genotype is go^1 pk^ + ro^1 and its phenotype is golden cuticle at all stages and rose eye from larvae on (see also the Results section). The *Anopheles gambiae* mosquitoes, obtained weekly from LPD, NIAID, NIH, were employed on August 2000 at the Laboratory of Fundamental and Applied Cryobiology, University of Tennessee, Knoxville, TN, USA.

Larvae were reared at 26 ± 1°C in rectangular plastic basins (*Ae*. *aegypti*, *An*. *aquasalis* and *Cx*. *quinquefasciatus*) or rectangular iron pans coated with vitreous enamel (*An*. *quadrimaculatus*) containing 300 specimens within 1 liter of water and with 1 gram of food being provided every two days. Water and diet source varied according to the mosquito species: dechlorinated water and cat food Friskies (“Peixes–Sensações marinhas”, Purina, Camaquã, RS, Brazil) for *Ae*. *aegypti* and *Cx*. *quinquefasciatus*, brackish dechlorinated water (2 mg of marine salt/mL of dechlorinated water) and fish food Tetramin (*Tetramarine Saltwater Granules*, *Tetra GmbH*, *Germany*) for *An*. *aquasalis*, tap water and brewer’s yeast/liver powder (1:1) for *An*. *quadrimaculatus*. In all cases, adults were kept at 26 ± 1°C, 12/12 h light/dark cycle, 70–80% relative humidity and fed *ad libitum* with 10% sucrose solution, except when prepared for blood feeding (see below).

### Synchronous egg laying

The synchronous egg laying method was adapted from Valencia *et al*. [26,27], as previously described [17,19,20]. For egg production, females of all species, three to seven days old, were sugar deprived for 24 hours and then blood-fed on anaesthetized chickens (*An*. *quadrimaculatus*) or guinea pigs (all other species). Immediately before egg laying induction, females were transferred to 15 mL centrifuge tubes and anesthetized in ice for a few minutes. The interval between blood meal and egg laying induction, as well as the procedure adopted for obtaining eggs, varied according to the species.

*Aedes aegypti* and all anopheline females were anaesthetized in ice three to four days after blood feeding. Groups of five to ten anaesthetized females were then rapidly transferred to upside down 8.5 cm diameter Petri dishes, where the lid became the base. This base was internally covered with Whatman No. 1 filter paper. After the females regained activity, a process that took 3–10 minutes, the filter paper was soaked with the same water employed to rearing each species, thus stimulating the laying of the eggs that were deposited individually or in small disorganized groups. In the case of *An*. *gambiae*, females also laid eggs on filter paper soaked in an aqueous solution containing 100 μM of L-Ascorbic Acid (Sigma # A92902) and 500 μM of Benserazide (DL-Serine 2-(2,3,4-trihydroxybenzyl)hydrazide) (Sigma # B7283). This solution was prepared immediately before use and kept in the dark during all the procedure. Benserazide inhibits DDC thus blocking the process of melanization (Fig 1B) [9] while ascorbic acid prevents benserazide oxidation.

Groups of five to ten *Cx*. *quinquefasciatus* females were anaesthetized in ice five to six days after the blood meal and then transferred to 8.5 cm diameter Petri dishes in the normal position (not upside down) without filter paper. After insect recovery, dechlorinated water was added with the aid of a micropipette through a small hole in the lid until the females were pressed against it, which stimulated egg laying. A second small hole was present in the lid to allow air outlet while water was being introduced. Eggs were deposited in organized rafts containing from few dozens to hundreds of eggs.

In all cases egg laying lasted one hour in the dark, inside an incubator at 25 ± 1°C. Petri dishes were then opened inside a large cage where the females were released. Eggs were allowed to develop at 25°C until being employed in the experiments. For *Ae*. *aegypti* and anopheline eggs the sides of the Petri dishes were sealed with parafilm, in order to avoid water evaporation. For *Cx*. *quinquefaciatus* eggs, rafts were kept intact prior to the first experimental point, when they were transferred to Petri dishes whose base was covered with Whatman No. 1 filter paper soaked with dechlorinated water. Rafts were carefully disrupted and the eggs were spread with the aid of a painting brush.

### Ethics statement

The procedure and use of live chicken followed the UF-IACUC Protocol no. 201003892. The procedure and use of anaesthetized guinea pigs was reviewed and approved by the Fiocruz institutional committee ‘Comissão de Ética no Estudo de Animais’ (CEUA/FIOCRUZ), license number: L-011/09.

### Eggshell darkening analysis in *Ae*. *aegypti*, *An*. *aquasalis* and *Cx*. *quinquefasciatus*

Eggs at approximately 80% of embryogenesis completion had their exochorion removed with bleach (NaOCl, 6% active chlorine) treatment for one minute followed by three washes with dechlorinated water and were kept in moist filter paper until hatching. These exochorion-depleted eggshells (i.e. composed of an outer endochorion and an inner serosal cuticle) were then transferred into a microscopy slide and bright field images were obtained with a digital imaging acquisition system coupled to a Zeiss Axio Scop 40 microscope. Two experiments per species were performed, each one consisting of at least 9 eggshells. The image acquisition setup was the same, in both the microscope and the computer, for all images. Eggshell melanization degree was evaluated employing the ImageJ software (https://imagej.nih.gov/ij/) with the 'Measure' function within the 'Analyze' menu. This function calculates the mean densitometric value of the selected area in an 8-bit grey scale, i.e. a completely white and a completely black pixel have, respectively values of 255 and 0. Representative circular regions were selected, always close to the hatching line. The densitometry of each eggshell was subtracted against the densitometry of a fixed circular region of non-saturated white background (with a value of 232). Densitometry values were then inversed (i.e. a white and a black pixel measuring, respectively, 0 and 255) and darkening percentages were calculated, assuming the mean value of *Ae*. *aegypti* eggshells as 100%.

### Detection of serosal cuticle formation in *An*. *quadrimaculatus*

Serosal cuticle synthesis was evaluated in both WT and GORO strains of *An*. *quadrimaculatus* employing two approaches: air drying and bleach treatment, as previously described for the other mosquitoes [17–19].

For the air-drying assay, eggs at distinct stages of embryogenesis (comprising seven time points in total, see the egg shrinkage experiment shown on the Results section '*An. quadrimaculatus* GORO embryogenesis is normal, despite its impaired melanization' for details) were blotted onto a dry Whatman No. 1 filter paper to remove all water. Eggs were then left drying on air for 15 minutes, when shrunken or intact eggs were counted under a stereomicroscope. For each time point and each strain, three independent experiments were performed, each replicate consisting of 30 synchronized eggs. Experiments were performed at 25°C and the relative humidity varied between 65 and 75%.

Incubation with bleach for several minutes digests both the egg exochorion and endochorion while leaving the serosal cuticle intact. Synchronized *An*. *quadrimaculatus* eggs from both strains were treated with bleach (6% active chlorine) during 3–10 min at different stages of embryogenesis, before and after the abrupt change in egg permeability (detected through the air-drying experiment described above). The resulting material was analyzed under a stereomicroscope (MIA 3XS S/N 0342, Martin Microscope Company) with an Olympus U-CMAD3 U-TV1X 2 adapter and Nikon CodPix 5400 camera, coupled with a digital image acquisition system. For each strain and time point two independent experiments, each with at least 20 eggs, were performed.

### Definition of the end point of *An*. *quadrimaculatus* embryogenesis

The total period necessary for embryonic completion in both WT and GORO strains was defined as previously described for other mosquitoes [19,28]. Two hours before the (empirically) estimated hatching of the putative first larva, eggs were flooded with a solution of 150 mg/ 100 mL yeast extract (SIGMA # Y1625) prepared in tap water. Egg eclosion was counted hourly, until no more hatchlings were observed. Twenty four hours after the eclosion of the last putative larvae the samples were checked again to confirm that total hatching was recorded. The embryogenesis end point was defined as the period necessary to hatch 50% of total larvae. For each strain, three independent experiments, each with 120 eggs, were performed.

### Embryo viability under dry conditions

Except for *An*. *gambiae*, all species and strains were employed in this experiment. In each case, groups of 40 or 50 synchronized eggs, obtained as explained above (see above the Methods section 'Synchronous egg laying'), were removed from water and blotted onto dry Whatman N° 1 filter paper with the aid of a paint brush, at specific moments of embryogenesis (see below the Results section 'Egg resistance to desiccation after serosal cuticle formation is enhanced by melanization' for details). Eggs remained developing in this dry environment for 2, 5, or 10 hours. After these periods, eggs were transferred back to moist conditions until embryogenesis completion. In all experiments the total test interval ("wet-dry-wet") was shorter than the period necessary for embryogenesis completion. Egg viability was quantified through larval hatching, induced with 150 mg/ 100 mL yeast extract solution [19,28], prepared with the same water used for larvae rearing (see above the Methods section 'Mosquito sources and rearing'). Larval eclosion was recorded hourly until no more hatchlings were observed for two successive hours. Total larval hatching was confirmed 24 hours later.

Viability control samples containing at least 120 eggs, kept continuously in moist filter paper until the end of embryogenesis, were employed in all cases. Experimental data were normalized with these controls, whose hatching was induced with yeast extract solution (150 mg/ 100 mL).

Three independent experiments were performed for each species or strain, using triplicates at least, inside an incubator at 25±1°C. Relative humidity varied between 60 and 80% for both *An*. *quadrimaculatus* strains and between 20 and 55% for all other species.

### Statistical analysis

For the analysis of eggshell darkening, air drying, embryogenesis period and embryonic viability under dry conditions the adequate sample size (n) of each experiment was defined from preliminary experiments. For all these experiments, eggshells or eggs were randomly collected from the filter paper (see above the Methods section 'Synchronous egg laying'). Outliers were removed after Dixon's Q test. Kruskal-Wallis Nonparametric Test (P< 0.0001) was used in eggshell melanization analysis, One Way Analysis of Variance (ANOVA) followed by Tukey’s Multiple Comparison Test (P< 0.05) was used in the egg viability experiments and the Student’s t-test (P <0.001) was used to compare viability between the two *An*. *quadrimaculatus* strains. All statistical analyzes, except Dixon's Q test, were made using GraphPad Prism version 5.00 for Windows (GraphPad Software, San Diego, California USA, www.graphpad.com).

## Results

### Levels of eggshell melanization and egg resistance to desiccation (ERD) are directly related

**Fig 2:**
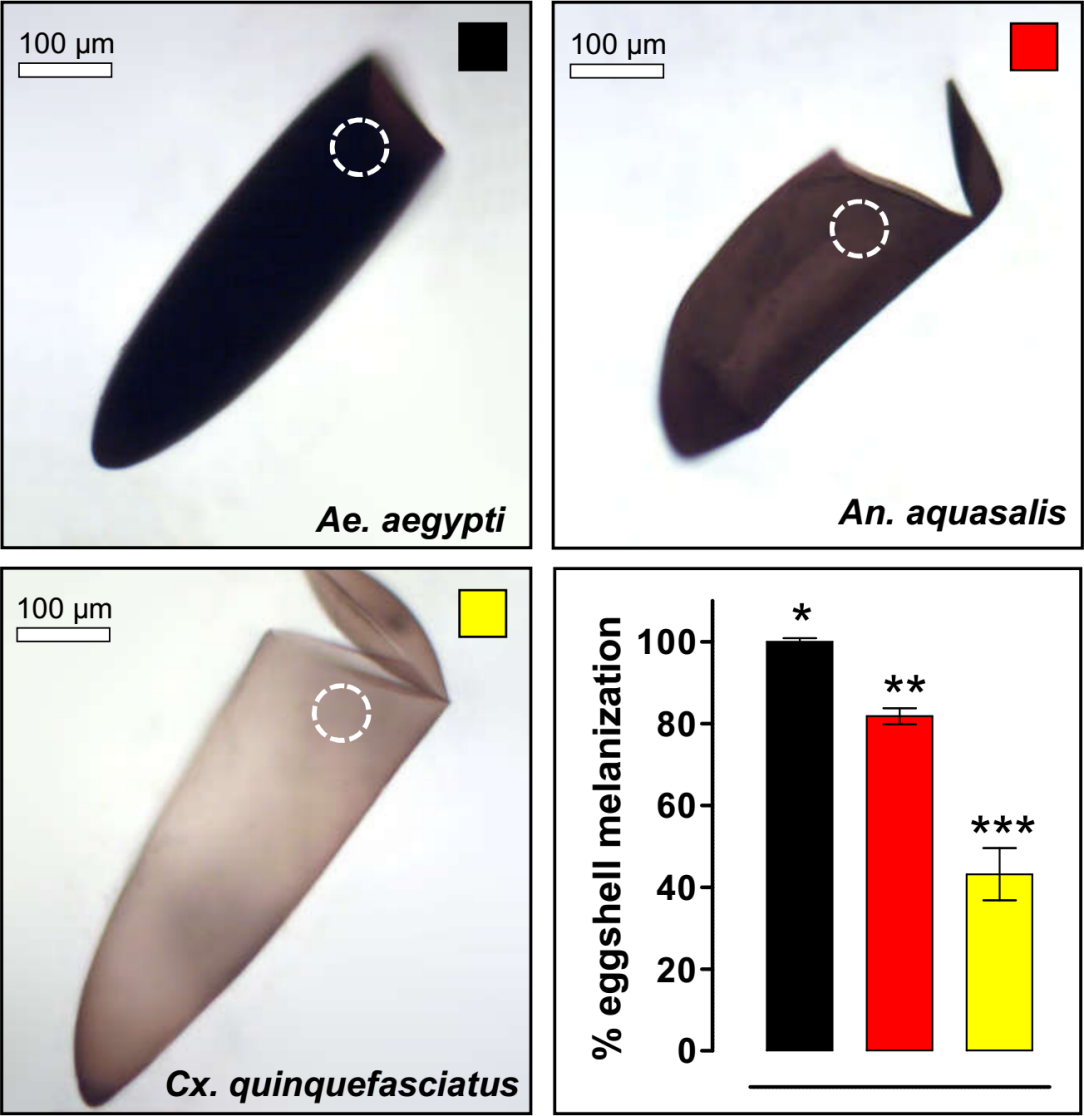
Mosquito eggshell melanization varies among species. Melanization degree was quantified in empty eggshell images obtained with bright field microscopy employing the ImageJ software (lower right graphic). The maximum melanization level was arbitrarily attributed to *Ae*. *aegypti* eggshells. The measured region, always near the hatching line, is indicated by dashed white circles. A direct correlation between melanization and ERD degree occurs (compare with Fig 1). Values represents the mean ± s.d. of two experiments, each consisting of at least 9 eggshells. All observed differences are statistically significant (Kruskal-Wallis, P < 0.0001).

The ERD, defined as the capacity of an egg to sustain its viability outside the water [29,30], varies among mosquito species at the end of embryogenesis (Fig 1C) [19]. In order to evaluate if these viability differences could be explained by egg pigmentation, the degrees of melanization of hatched eggshells (without exochorion, see Methods) of *Ae*. *aegypti*, *An*. *aquasalis* and *Cx*. *quinquefasciatus* were assessed (Fig 2). Eggs of *Ae*. *aegypti* and *An*. *aquasalis* present a homogeneous pigmentation, while *Cx*. *quinquefasciatus* eggs are more pigmented near its extremes. In spite of this, *Ae*. *aegypti* exhibits the greater eggshell pigmentation, followed by *An*. *aquasalis* and *Cx*. *quinquefasciatus*.

Although establishing a direct relationship between eggshell pigmentation and ERD is tempting, other eggshell related factors, such as differences in thickness or components of the endochorion or the serosal cuticle, might account for this distinctness [1,2,9,20,31]. Moreover, since we are studying mosquitoes of different genera, whose common ancestor occurred ~217 million years ago [32], embryological and egg traits vary considerably [19,20] and may not be comparable (see Discussion). In order to directly evaluate the relationship between melanization and ERD without any other confounding factor, we took advantage of a mutant strain of the species *An*. *quadrimaculatus*, which shows a significant melanization deficit: the GORO strain.

### *An*. *quadrimaculatus* GORO embryogenesis is normal, despite its impaired melanization

**Fig 3:**
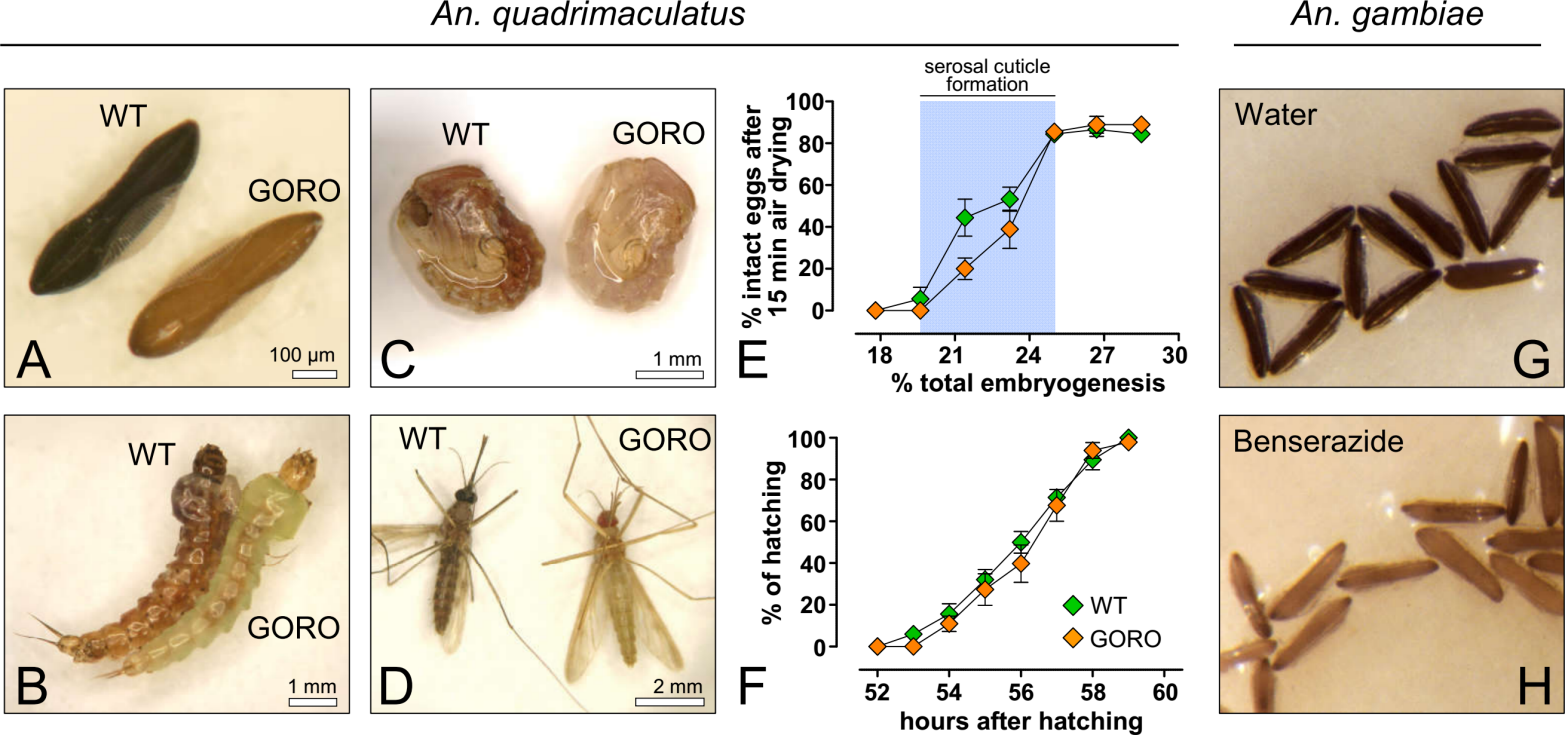
Embryogenesis of the weakly pigmented *Anopheles quadrimaculatus* GORO strain proceeds similarly to the WT. GORO means ‘GOlden cuticle and ROse eyes’. (**A**) eggs, (**B**) larvae, (**C**) pupae and (**D**) adults. (**E**) Eggs at different embryonic ages developing at 25°C were air-dried for 15 minutes and the percentage of eggs that did not shrink (i.e. intact eggs) was then registered. Relative humidity ranged between 65 and 75%. The abrupt alteration in egg permeability is coupled with serosal cuticle formation, highlighted by a blue stripe (see Fig 4). Each lozenge represents mean ± s.e. of three independent experiments, each one with 30 eggs per time point (total of 630 eggs per strain) (**F**) Cumulative larval hatching at 25°C; data were normalized by total eclosion, obtained 24 hours after the expected embryogenesis completion. Each curve represents mean and standard error of three independent experiments consisting of 120 eggs each (total of 360 eggs per strain). (**G**, **H**) The lack of proper melanization can be phenocopied in the mosquito *An*. *gambiae*: while eggs laid in water become dark-brown (**G**), those laid on a benserazide solution, a melanization inhibitor (Fig 1B) develop a golden color (**H**).

**Fig 4:**
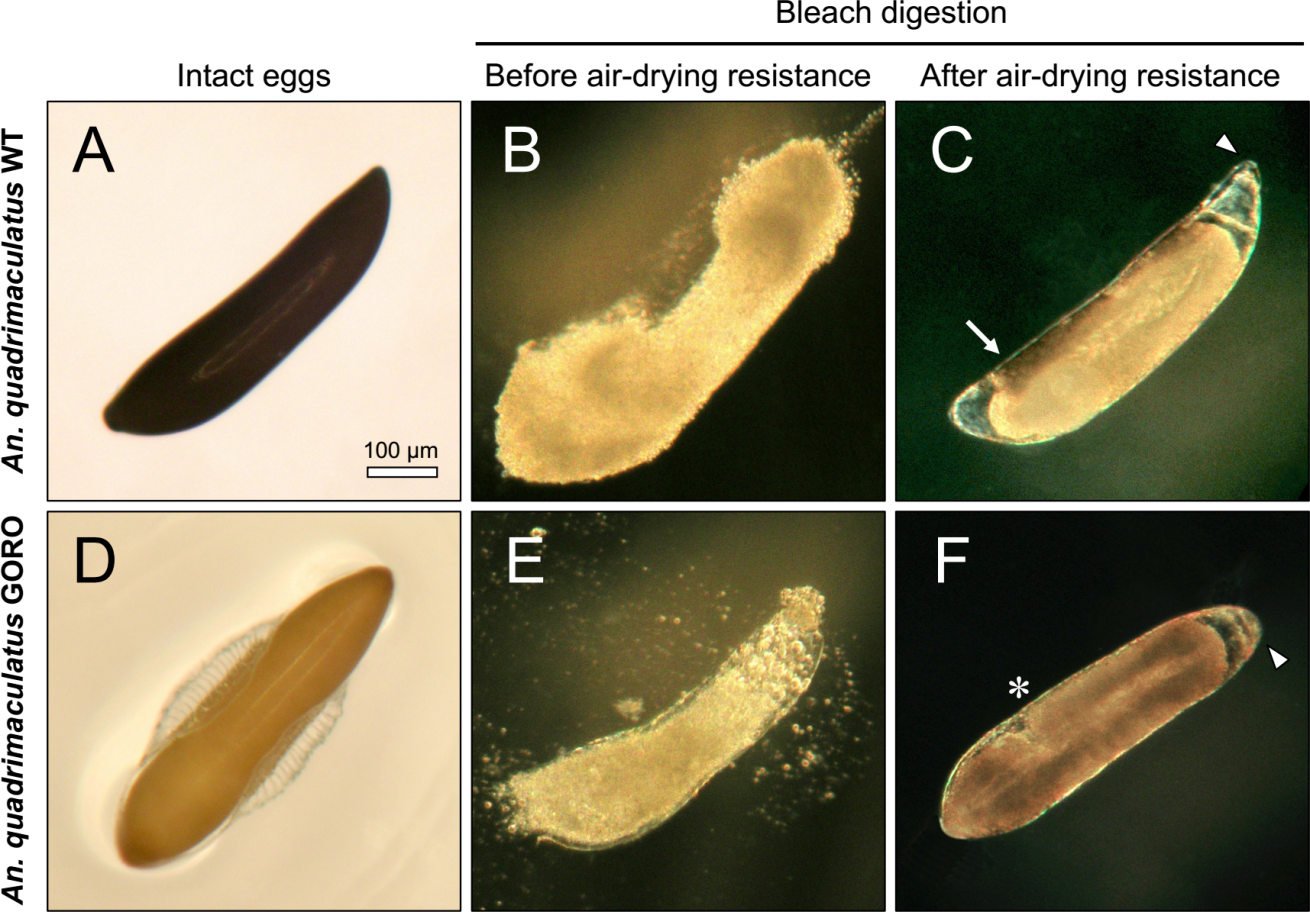
Resistance to air-drying is related to serosal cuticle formation in both *An*. *quadrimaculatus* strains. Serosal cuticle presence was determined by chorion digestion driven by bleach (6% active chlorine). (**A**, **D**) Intact eggs. (**B**, **E**) Eggs treated with bleach before acquisition of air-drying resistance are totally digested while (**C**, **F**) eggs exposed to the same procedure after acquisition of air-drying resistance remain intact due to the presence of the serosal cuticle (see Fig 3E). Arrow: endochorion remnants not yet digested; arrowheads: serosal cuticle boundaries; asterisk: posteriormost end of the germ band. All images are in the same magnification.

The mosquito *An*. *quadrimaculatus* is endemic to the Eastern part of North America, being a primary vector of malaria in this region [33]. The wild type strain of this species presents a dark-brown, melanized eggshell and a dark-brown cuticle in larval, pupal and adult stages (Fig 3A–3D). On the other hand, the GORO strain carries a *golden cuticle* mutation, which causes poor body melanization in all life stages [34], within a *rose eye* background (see Methods), (Fig 3A–3D). In order to assess whether the lack of proper melanization compromises embryogenesis, two embryonic traits were analyzed in WT and GORO: the chronology of serosal cuticle formation and the completion of embryogenesis (Fig 3E and 3F and Fig 4). Serosal cuticle formation, assessed through the abrupt acquisition of resistance to egg shrinkage (Fig 3E) and bleach digestion (Fig 4), as previously described in other mosquito species [17,19], occurs in between 19.6 and 25% of total embryogenesis, at the stage of complete germ band elongation (Fig 4), in both strains. Likewise, the period necessary for entire embryogenesis, approximately 56 hours after egg laying, is similar in both strains (Fig 3F), as well as the viability percentage (mean ± s.d.): 58.1 ± 4.7% for WT and 53.3 ± 8.8% for GORO. To confirm that the dark pigment of *Anopheles* eggs is due to the production of melanin, and not of other pigment, eggs of *An*. *gambiae* were laid on water or on a benserazide solution, an inhibitor of DDC [9], that participates in the melanization pathway (Fig 1B). While eggs laid on water turn from white to dark-brown (Fig 3G), those laid on the benserazide solution turn from white to yellow (Fig 3H), phenocopying the GORO mutation of *An*. *quadrimaculatus*. Therefore, the lack of melanization in the *An*. *quadrimaculatus* GORO mutant does not compromise neither serosal cuticle formation nor the total period necessary for embryogenesis completion.

### Egg resistance to desiccation after serosal cuticle formation is enhanced by melanization

**Fig 5:**
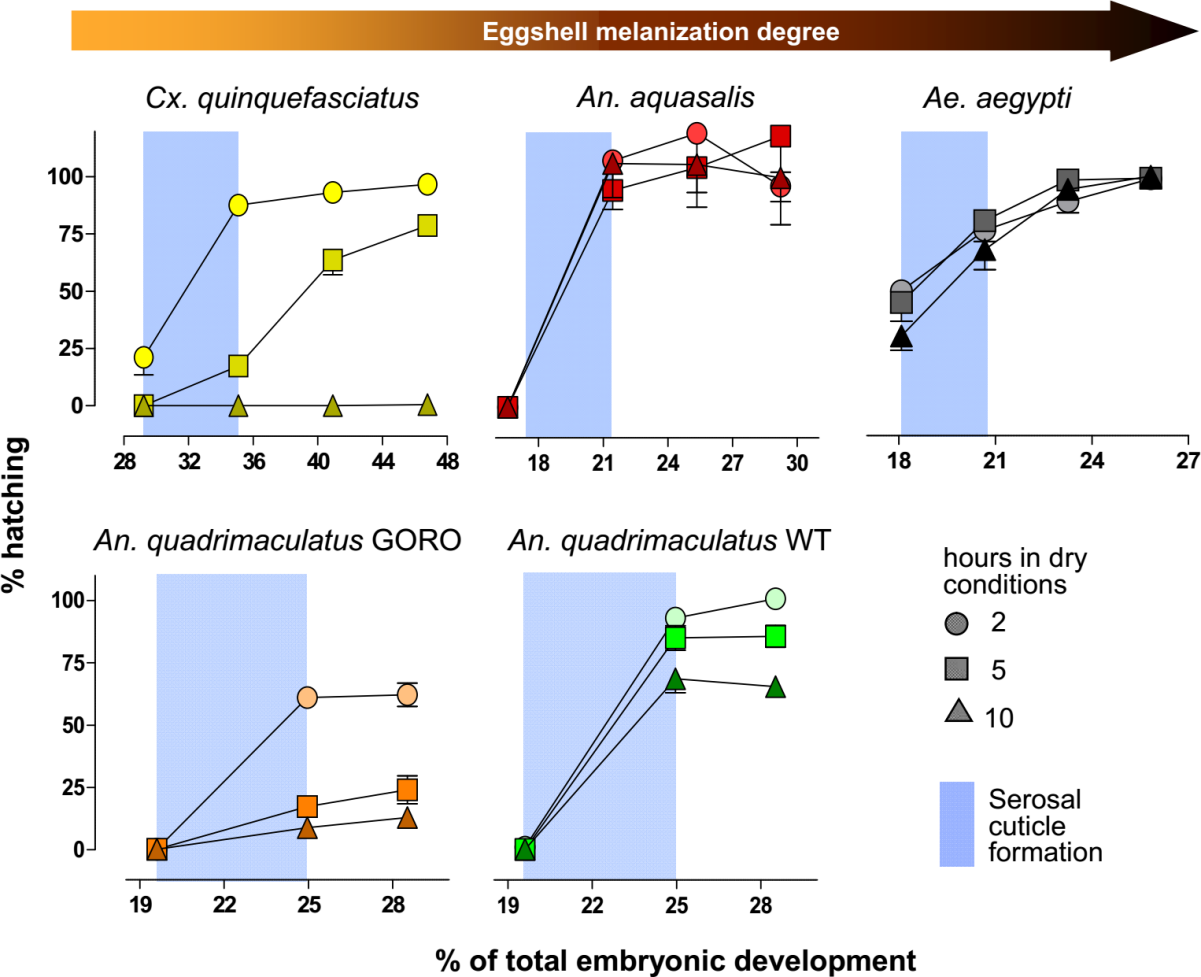
Mosquitoes with darker eggshells resist more to desiccation. Mosquito eggs were laid on water. Values in the *x*-axis indicate the moment that eggs were transferred to dry conditions, staying outside the water for 2, 5 or 10 hours. Eggs were then returned to moist filter paper until completion of embryo development, when hatching rates were evaluated. Data were normalized regarding to control samples, kept on moist conditions throughout development. Blue stripes indicate the serosal cuticle formation period (as shown in Figs 1 and 3). Each point represents mean ± s.e. of three independent experiments consisting of at least 120 eggs each. A total of at least 3,240 eggs were employed for each species or strain. In all cases viability was significantly different between the two first experimental points (i.e. before and after serosal cuticle formation) (ANOVA followed by Tukey’s test, P < 0.05, see Table 1); the exception being *Cx*. *quinquefasciatus* at 10 hours in dry conditions. After serosal cuticle formation, *An*. *quadrimaculatus* GORO eggs were less viable than WT ones under equivalent conditions, in all cases (Student’s t-test, P < 0.001). All experiments were conducted at 25°C and relative humidity of 60–80% (*An*. *quadrimaculatus*) or 20–55% (other species).

The interspecific difference in egg viability when these are placed outside the water at late embryogenesis (Fig 1C) [19] might be due to other factors, unrelated to the eggshell and its serosal cuticle. For instance, it could be caused by specific metabolites inside the egg or present in the pharate larvae, such as glycerol, trehalose, glycogen or triacylglycerols, or to significant variation in the larval cuticle structure [29,30,35–37]. Thus, we uncoupled serosal cuticle participation in ERD from other factors. Eggs from the different mosquito species and strains were removed from the water at different stages of early embryogenesis and left developing outside the water for two, five or ten hours. Hatching rates were assessed at the end of embryogenesis (Fig 5 and Table 1). In all cases serosal cuticle formation significantly increases egg viability outside the water (ANOVA followed by Tukey’s Multiple Comparison Test, P < 0.05). The role of the serosal cuticle on ERD of *Ae*. *aegypti* left up to ten hours in a dry environment is partial: the serosal cuticle elevates embryo viability from 30–50% before its formation to 68–81% right after its synthesis. However, all *Ae*. *aegypti* eggs die if remaining outside the water for 25 hours prior to serosal cuticle formation [17]. In *Anopheles* species and strains the serosal cuticle formation is essential: egg viability in dry conditions is null before, but increases considerably after serosal cuticle synthesis, as previously described for *An*. *quadrimaculatus* [38] and *An*. *gambiae* [18]. In both *Ae*. *aegypti* and *An*. *aquasalis* the hatching rate in each stage is equivalent for all dry exposure periods. Regarding *Cx*. *quinquefasciatus*, 20% of the eggs left outside the water for two hours before serosal cuticle synthesis survive but similar aged eggs exposed to a dry environment for longer periods do not. Moreover, egg viability after serosal cuticle formation is inversely proportional to the exposure period outside the water. Interestingly, in both *Cx*. *quinquefasciatus* and *Ae*. *aegypti*, a gradual increase in embryo viability was observed after serosal cuticle formation, suggesting this structure follows a process of maturation until it becomes completely functional. Regarding *An*. *quadrimaculatus*, in both strains the percentage of viable eggs is inversely associated with the dryness period. In all conditions after serosal cuticle formation, GORO eggs are far more sensitive to dehydration than wild type ones (Student’s t-test, P < 0.001). For instance, at 25% of total embryogenesis and when left for 5 hours in a dry environment, the hatching rate of WT and GORO strains are, respectively, 85 and 17%.

**Table 1:** Egg viability of mosquito species and strains under dry conditions during embryogenesis, before and after serosal cuticle (SC) formation.

Species or strain	Hours under dry conditions	Stage of embryogenesis^#^			
		Before SC formation	After SC formation I	After SC formation II	After SC formation III
*Ae*. *aegypti*	2	50.0 ± 32.4 ^a^	76.4 ± 16.9 ^b^	88.9 ± 16.8 ^b^	98.9 ± 11.5 ^b^
	5	44.9 ± 28.3 ^a^	80.6 ± 10.5 ^b^	98.3 ± 15.6 ^b^	99.1 ± 14.1 ^b^
	10	30.2 ± 21.3 ^a^	67.9 ± 30.4 ^b^	94.3 ± 13.4 ^c^	100.0 ± 17.9 ^c^
*An*. *aquasalis*	2	0.0 ± 0.0 ^a^	107.8 ± 33.4 ^b^	119.7 ± 56.7 ^b^	96.7 ±11.5 ^b^
	5	0.0 ± 0.0 ^a^	94.8 ± 24.9 ^b^	104.8 ± 52.2 ^b^	118.6 ± 47.3 ^b^
	10	0.0 ± 0.0 ^a^	106.6 ± 44.6 ^b^	106.2 ± 36.8 ^b^	100.3 ± 31.1 ^b^
*Cx*. *quinquefasciatus*	2	21.1 ± 22.8 ^a^	87.6 ± 12.3 ^b^	93.1 ± 10.2 ^b^	96.7 ± 7.3 ^b^
	5	0.0 ± 0.00 ^a^	17.4 ± 8.8 ^b^	63.7± 19.4 ^c^	78.7 ± 10.3 ^c^
	10	0.0 ± 0.0 ^a^	0.0 ± 0.0 ^a^	0.0 ± 0.0 ^a^	0.5 ± 0.9 ^b^
*An*. *quadrimaculatus* WT	2	1.0 ± 2.9 ^a^	92.9 ± 11.4 ^b^	106.6 ± 10.2 ^b^	N.D.
	5	0.0 ± 0.0 ^a^	84.8 ± 14.9 ^b^	85.5 ± 13.1 ^b^	
	10	0.0 ± 0.0 ^a^	68.5 ± 16.9 ^b^	65.3 ±11.3 ^b^	
*An*. *quadrimaculatus* GORO	2	0.0 ± 0.0 ^a^	60.9 ±6,4 ^b^*	62.0 ± 14.0 ^b^*	N.D.
	5	0.0 ± 0.0 ^a^	17.2 ± 9.2 ^b^*	23.9 ± 16.8 ^b^*	
	10	0.0 ± 0.0 ^a^	8.7 ±7.4 ^b^*	12.8 ±7.1 ^b^*	

^#^ The stages of embryogenesis are indicated in the *x*-axis of Fig 5.

Values represent mean and standard deviation of at least three independent experiments for each species and period under dry conditions. Every experiment employed a total of at least 120 eggs for each point and for each species or strain. Hatching percentages were normalized according to control samples kept moist throughout development.

^a, b, c^ Different letters represent significant differences among the distinct stages of embryogenesis in the same drying period and for the same species or strain (ANOVA, followed by Tukey’s test P<0.05).

* Asterisk means significant differences between *An*. *quadrimaculatus* WT and GORO strains in the same drying period and for the same stages of embryogenesis (Student’s t-test, P <0.001).

N.D.: Not determined.

In this assay the viability of the control samples kept moist throughout development was as follows (mean ± s.d.): 65.8 ± 8.0 for *Ae*. *aegypti*, 87.1 ± 8.6 for *Cx*. *quinquefasciatus*, 64.9 ± 15.3 for *An*. *aquasalis*, 59.2 ± 4.7 for *An*. *quadrimaculatus* WT and 56.7 ± 10.2 for *An*. *quadrimaculatus* GORO.

The increased eggshell susceptibility to water loss of the *An*. *quadrimaculatus* mutant was further confirmed via a collapsing experiment employing ethylene glycol, a cryoprotectant [26,27]. In this experiment, the strain employed was the MRA-123 (GOCUT), containing only the mutation with the *golden cuticle* phenotype, without the mutation of the rose eye phenotype. In the presence of ethylene glycol, *An*. *quadrimaculatus* GOCUT eggs loose water faster than *An*. *quadrimaculatus* WT ones (Denise Valle, personal communication). This experiment also shows that the higher water loss susceptibility is associated only with the lack of proper melanization, having no relation with the eye pigment mutation.

## Discussion

### Regarding the *Anopheles quadrimaculatus* GORO strain

The existence of the *An*. *quadrimaculatus* GORO strain allowed to demonstrate that egg resistance to desiccation in mosquitoes is heavily dependent on serosal cuticle formation and, at the same time, that eggshell melanization positively impacts the egg survivorship outside the water. Although this interesting strain exists for at least 16 years [34], this is the first peer-reviewed report employing GORO. The genetics of the *golden cuticle* mutation (GOCUT) present in the *An*. *quadrimaculatus* GORO is currently unknown. The enzymes N-β-alanyldopamine hydrolase (*tan*) and DCE (*yellow*), both present in the melanization pathway [15] (see also Fig 1B) do not seem to be related to the GORO mutant as determined by biochemical assay (Paul Howell, personal communication). Despite this, we worked on a development window within which the parameters of physiology and viability that are relevant to our biological question are equivalent between the wild *An*. *quadrimaculatus* and the GORO mutant. Given that melanization is also associated with immunity, it would be interesting to evaluate how the GORO strain responds immune challenges in adults, larvae and eggs [39,40].

It is worth mentioning that it would not be possible to use the same approach, at least with *Aedes* mosquitoes: the mutants *bronze* and *gray*, presenting altered egg color, are embryonic lethal [41], as well as gene silencing for *Laccase 2*, whose white eggs never darken [14]. In addition, the administration of α-MDH ((DL)-3-(3,4-dihydroxyphenyl)-2-hydrazino-2-methylpropionic acid, also named D,L Carbidopa) or benserazide, inhibitors of DDC activity, impedes eggs to darken completely, rendering tanned eggs (i.e. with a yellow/golden color, similar to GORO eggs); however these less melanized eggs are not viable [12,42]. Sometimes these non-melanized, non-sclerotized eggs burst [14] most likely due to the fragile eggshell that does not bear the amount of water absorbed. Since Laccase2 and DDC are in the melanization pathway (Fig 1B), the fact that their absence impedes mosquito eggs to darken shows that this dark pigment is due to the production of melanin.

### The role of egg color in insects

Insect eggs occur in a myriad of colors, ranging from white to black with tones of yellow, orange, red, pink, green and brown, among others. Egg color may occur uniformly or in patches throughout the eggshell, or can appear in restricted areas [35]. These colors are produced by pigments such as melanins, sclerotins, ommochromes, pteridines, carotenoids and flavonoids [23,43].

Egg colors are associated with defense strategies against predators, such as homochromy, mimicry, camouflage, visual disruption and warning (aposematic) signaling [35]. Females of the bug *Podisus maculiventris* selectively control egg color during oviposition: darker and lighter eggs are laid on the upper and lower surface of leaves, respectively. The dark pigment protects eggs against the deleterious effects of UV light emitted from the sun [44].

This list is further expanded with melanin participation in the egg resistance to desiccation (ERD). The ERD trait has been associated with the staggering adaptive success insects show on land [45,46]. Two questions arise from the above considerations. A direct exposition to sunlight also increases evaporation of eggs [35]: does the dark pigment selectively present in eggs of *P*. *maculiventris* also protects against desiccation? In relation to the other non-melanin pigments; do they also protect insect eggs and cuticles in post-embryonic life stages from water loss?

### Melanin and desiccation resistance in adult insects

The melanin contribution for desiccation resistance has been previously described in adult insects: Kalmus [22] compared the desiccation resistance in adults of wild type and *yellow*, *ebony* and *black* mutants of the *Drosophila melanogaster* fly. The wild type cuticle is melanized, the cuticle of *yellow* mutants is light brown/yellowish (i.e. with a tanned color) and the cuticle of *black* or *ebony* mutants is darker than wild type ones. The more melanized a fly is, the more it resists desiccation. The DCE/*yellow* gene is related to the activity of Dopachrome conversion enzyme, required for proper melanin formation. Both *black* and *ebony* genes code for enzymes necessary for NBAD production, driving dopamine usage for sclerotization, instead of melanization (Fig 1B); *i*.*e*. *black* and *ebony* mutants are defective in the sclerotization pathway and present a cuticle darker than the wild type one [15]. The same pattern was found in distinct species and morphs of *Hemideina* wetas from New Zealand and morphs of *D*. *melanogaster* from the Indian subcontinent: darker adults resist more against desiccation [24,25]. In the beetle *Tribolium castaneum* silencing of the gene *yellow-e* (*TcY-e*) leads to desiccation sensitivity of adults. These adults survive when reared at high humidity but, intriguingly, develop a slightly darker cuticle [47].

On the other hand, populations of *D*. *melanogaster* artificially selected for increased pigmentation do not resist desiccation more than control flies [48]. This apparent incoherence might be due to other factors, since the reduction in the rate of water loss by the cuticle is one out of the three aspects of the desiccation resistance (see below). Other explanation could be associated with the physicochemical properties of the melanin produced.

### How does melanin protect insect structures against desiccation?

Melanin might protect against desiccation due to its covalent or noncovalent interaction with other biomolecules such as proteins and chitin [15]. If this is the case, this association is distinct from sclerotization-driven crosslinking: both *black* and *ebony D*. *melanogaster* mutants are more melanized and present a cuticle that is less stiff and puncture-resistant (*i*.*e*. less sclerotized) than wild type ones [49]. Similarly, the elytral cuticle of *T*. *castaneum black* mutants are more viscous and less stiff than wild type ones [50].

Another hypothesis is that melanin might be hydrophobic and thus hamper water flux through the cuticle, as recently suggested [48]. Although both melanin precursors (DHICA and DHI, Fig 1B) are hydrophilic compounds, the molecular structure of melanin polymers varies depending on the biochemical conditions of polymerization and, therefore, "melanin" is a diffuse term for a rather diverse group of complex pigments [10,15,51–53]. In fact, there exists in the literature descriptions of melanin being both water-soluble [54] and water-insoluble [53]. Thus the *D*. *melanogaster* darker-selected populations might not have a higher desiccation resistance [48] due to the production of "hydrophilic melanins" in this specific situation.

A third hypothesis is that melanin might act decreasing the eggshell porosity, as suggested by experiments performed in fungus. In ascomycetes the melanin produced and deposited in the chitin-containing cell wall increases desiccation resistance [55]. This occurs, most likely, due to the decrease in the cell wall porosity conferred by melanin [56].

Independent of the mechanism, the eggshell of GORO eggs looses water more rapidly than the eggshell of WT eggs, as mentioned above.

In any case, although melanization in some instances increases desiccation resistance, as shown in the present work, this is not an universal rule [57], as exemplified below for other insect eggs.

### Melanin localization in the eggshell and other egg traits related to desiccation resistance

In any organism, an increase in resistance to desiccation is associated with three aspects: a higher initial body water store, a reduction in the rate of water loss and an increase in the tolerance to water loss [24,29,30,37]. Right after being laid on water, *Ae*. *aegypti* eggs promptly uptake water, increasing in weight and volume until the serosal cuticle is formed [21] and most likely the same happens with *Anopheles* and *Culex* eggs [20].

In mosquitoes, the role of eggshell in ERD is related to the reduction in the rate of water loss. The outermost mosquito eggshell layer is the exochorion, a delicate layer that easily detaches from the endochorion and does not participate in ERD [9,20]. Although the endochorion visibly melanizes, the serosal cuticle below it might also do so. In previous works our group has shown images of transparent serosal cuticles from different mosquito species [17–20]. However, these cuticles were obtained through bleach treatment, which digests the chorion. During this process, the bleach-resistant serosal cuticle might get unpigmented. In the mosquito *An*. *gambiae*, the serosal cells, which produce the serosal cuticle, express *tyrosine hydroxylase* and *dopa decarboxilase* genes [18], coding for enzymes related to both melanization and sclerotization pathways (Fig 1B) [15,23]. Beckel demonstrates that mosquito eggs without exo and endochorion exhibit a permeable serosal cuticle. Together with the known permeability of eggs before secretion of the serosal cuticle, it seems that the endochorion-serosal cuticle bonding is the functional entity responsible for reducing water loss [58]. This bonding would occur through crosslinking quinones derived from the sclerotization, through interactions with melanins [15,49], or both. In any case, the higher amount of water lost by a mosquito egg leads to a lower hatchability [21].

A moderate level of ERD before serosal cuticle formation was observed in *Ae*. *aegypti* and *Cx*. *quinquefasciatus*, but not in *Anopheles* spp.. This feature cannot be associated with the presence of melanin since it is observed in *Ae*. *aegypti* and *Cx*. *quinquefasciatus* that have opposite levels of eggshell melanization, while *Anopheles* spp. have intermediate levels. This viability might be due to an increased tolerance to water loss or a higher initial egg water content. Indeed, the percentage of eggshell weight in relation to total egg weight suggests that total body water content is lower in *An*. *aquasalis* [20].

Notwithstanding, color traits related to the decrease in water loss evolve differentially in other insect eggs. The eggshells of the cricket *Acheta domesticus* and the beetle *Tribolium castaneum* are transparent. In *A*. *domesticus* the molecules dopa, dopamine and NADA, (Fig 1B), are present in the serosal cells and cuticle most likely participating in the sclerotization pathway [59]. In *T*. *castaneum* the serosal cuticle is fundamental for ERD [46] and gene silencing of *Laccase2*, related to both melanization and sclerotization (Fig 1B) [15] diminishes the ERD level of this beetle [60].

### Evolution and ecology of resistance to desiccation in mosquito eggs

Mosquitoes of *Aedes*, *Culex* and *Anopheles* genera shared a last common ancestor ~217 million years ago. The subfamilies Culicinae (containing *Aedes* and *Culex* genera) and Anophelinae have separated ~204 million years ago [32]. Within this time span the level of pigmentation has greatly diverged, to the point where *Ae*. *aegypti* and *Cx*. *quinquefasciatus*, more closely related among themselves than *Anopheles* species, show the highest divergence in levels of eggshell pigmentation and desiccation resistance.

In mosquitoes, egg resistance to desiccation is a trait that guarantees survival in hostile environments and enables population growth and spread to new habitats [61,62]. In the case of *Ae*. *aegypti*, with a high ERD, this implicates in vector dispersion and promotes transmission of diseases such as chikungunya [6], dengue [7] and Zika [5]. Mosquito species with increased ERD are contained in a few genera (*Aedes*, *Haemagogus*, *Ochlerotatus*, *Opifex* and *Psorophora*), adding to about 30% of all described species [61].

**Fig 6:**
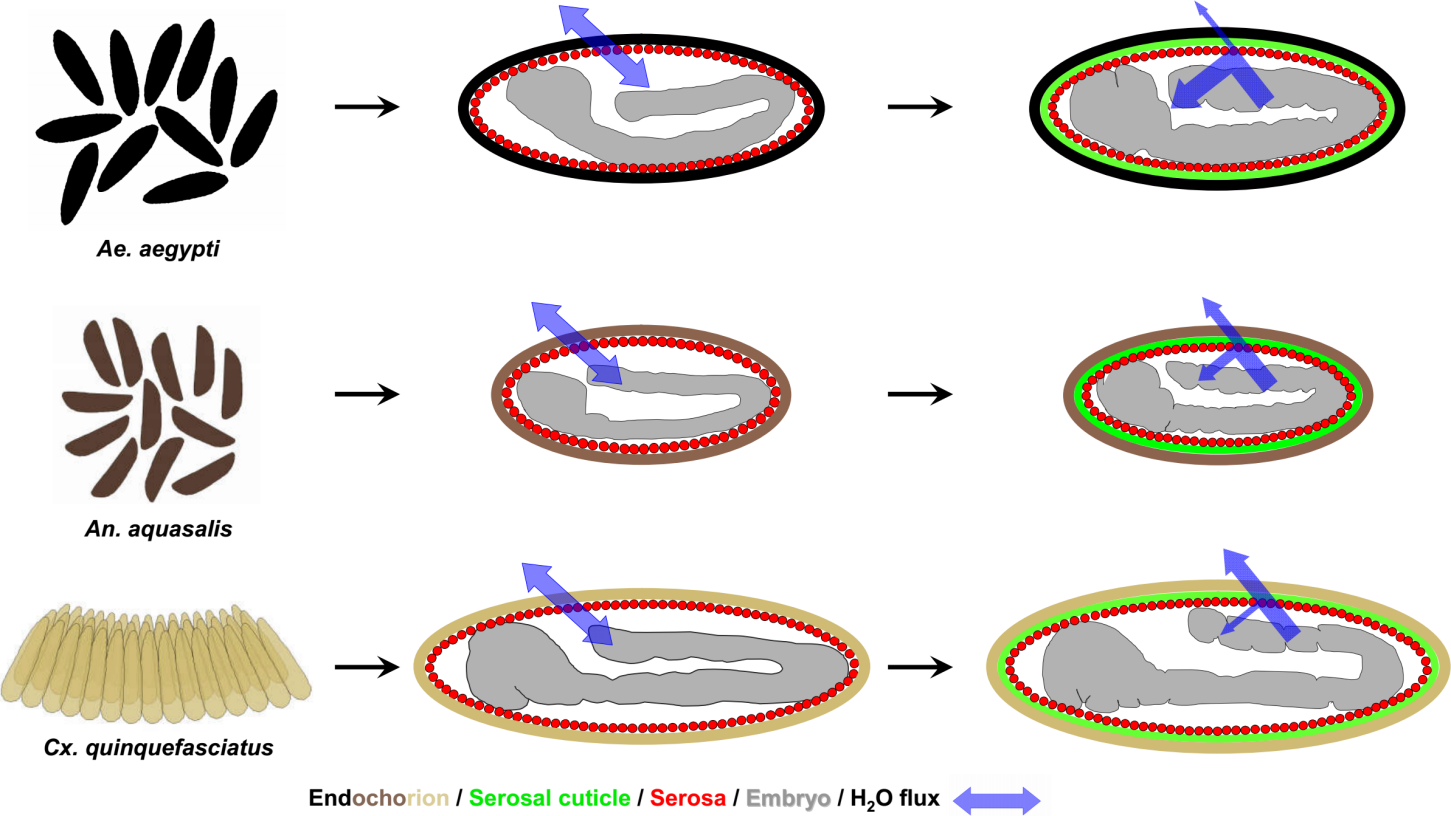
Mosquito vectors egglaying behavior and water flux through the eggshell before and after serosal cuticle formation. From top to bottom, leftmost panel: while *Ae*. *aegypti* and *An*. *aquasalis* females lay their eggs individually, the females of *Cx*. *quinquefasciatus* lay their eggs as an organized raft that floats on the water surface. In all species, before serosal cuticle formation water passes freely through the eggshell. Serosal cuticle formation diminished water passage through the eggshell in a color-dependent manner: while in *Ae*. *aegypti*, with a black endochorion, most of the water is retained inside the egg, in *An*. *aquasalis*, with a dark-brown endochorion, some of the water is retained inside the egg, but not all. Finally, in *Cx*. *quinquefasciatus*, with a light-brown/light-tanned endochorion, most of the water escapes and only a small portion of it is retained inside the egg. The depicted embryonic morphology are representative for each stage and species [19] and egg sizes among species are depicted in their natural proportion [20]. For the sake of simplicity, the outermost eggshell layer (the exochorion) and the other extraembryonic membrane (the amnion) are not depicted here. The exochorion does not participate in the ERD [20].

*Aedes aegypti* shows an outstanding success in keeping its eggs viable outside the water, up to 8 months in the dry [1,2]. There is even a report that shows hatching of *Ae*. *aegypti* eggs after 15 months, when kept at 9°C [63]. Indeed, more detailed analysis reveals this hatching success is directly related to higher relative humidity [21]. The present results show that the increased *Ae*. *aegypti* eggshell melanization is one of the traits responsible for the extremely high ERD seen in this species (Fig 6).

Although species from other genera such as *Culex* and *Anopheles* show a less striking ERD [2,19], this trait might still be relevant for survival, at least for Anopheline species. Eggs of *Anopheles* mosquitoes are viable on a dry surface for approximately one day after the end of embryogenesis (Figs 1C and 6) [19,38]. However, when left at humid soil, egg viability increases up to 7 and 18 days in *An*. *quadrimaculatus* and *An*. *arabiensis*, respectively [64–66]; other species resist for even longer periods [2]. Anopheline egg survival in soil is crucial for sustaining the mosquito life cycle during the dry season and thus the maintenance of malaria transmission [67–69]. Moreover, adults from species of the *An*. *gambiae* complex show distinct levels of resistance to desiccation [37,70]. As a future prospect, it would be interesting to evaluate if these species have distinct levels of melanization in their eggshells and adult cuticles.

Females of *Cx*. *quinquefasciatus* oviposit in rafts containing from few dozens to hundreds of eggs arranged along their longitudinal axis. Eggs internal to the raft structure bear sides protected by contact with other eggs; their anterior region contacts the water film, and the posterior tip is the only region in contact with the air [2,71]. Beyond being darker than other eggshell regions (Fig 2), the posterior tip is the only endochorion region whose surface is rough and irregular, similar to the whole endochorion of *Ae*. *aegypti* eggshells [20]. Given that *Culex* eggs at raft edges were found dead after exposure to strong dry winds [2], it seems that the raft per se can act as a protection against dehydration, according to the egg cluster-desiccation hypothesis [72]. This could relax the selection pressure of other traits related to ERD, such as serosal cuticle efficiency and eggshell pigmentation, with the exception of the posterior tip. The occurrence of a higher rate of water loss through the *Culex* eggshell might be advantageous, in the context of a more efficient gas exchange and a increased defense against pathogens, as previously discussed [19].

In summary, eggshell melanization and serosal cuticle formation increase egg protection against water loss (Fig 6). However, we do believe that the differential egg resistance to desiccation observed in distinct mosquito species is a trait with multifactorial origins. For instance, among *Aedes*, *Anopheles* and *Culex* species, there are differences regarding egg size, volume, surface area and weight, eggshell surface density and weight, endochorion surface aspect and also a tendency in differences regarding eggshell chitin content [20]. Endochorion thickness also varies among species [1,2,9,31] as well as the embryonic stage of serosal cuticle formation and the total period of embryogenesis [19]. Therefore other factors might also contribute such as the thickness and texture of the distinct eggshell layers and the parental investment, observed in *Culex* species.

## Conclusions

Our results demonstrate that, in mosquitoes, the eggshell melanization level is directly associated with egg viability outside the water after serosal cuticle formation. Decoding the association between egg coloration and resistance to desiccation is relevant for studies concerning ecology and evolution of mosquitoes and other insects. Since eggshell and adult cuticle pigmentation ensure survivorship for some insects, they should be considered regarding species fitness and also for the control and management of vector or pest insects [73,74].
